# Management of Recurrent Giant Cell Tumor of Distal Tibia With Intramedullary Hindfoot Fusion Nail and Trabecular Metal Implant Construct

**DOI:** 10.7759/cureus.57922

**Published:** 2024-04-09

**Authors:** Kylie T Callan, Amanda Anderson, Ryan Kim, Amanda Goldin, Naudereh Noori

**Affiliations:** 1 Orthopedic Surgery, University of California Irvine Medical Center, Orange, USA

**Keywords:** tumor recurrence, foot and ankle reconstruction, distal tibia, ankle arthritis, tibiotalocalcaneal nail, giant-cell tumor of bone, aneurysmal bone cyst

## Abstract

Reconstruction options for giant cell tumors (GCTs) of bone are limited and challenging due to the amount of structural compromise and the high recurrence rates. This is especially true for GCTs of the foot and ankle, as the area is vital for weight bearing and function. The typical treatment for GCTs is currently excision, curettage, and cementation, although that is not always effective. A 36-year-old otherwise healthy female presented with an original diagnosis of a large aneurysmal bone cyst (ABC) of the distal tibia that had recurred despite two previous attempts at treatment with resection and cementation. She was treated with surgical resection of the lesion, reconstruction, and ankle and subtalar joint arthrodesis with a tibiotalocalcaneal intramedullary nail in combination with a trabecular metal cone. The final pathology of the intraoperative samples was consistent with GCT. Postoperatively, she recovered well, and her imaging was consistent with a successful fusion. This case report provides evidence that tibiotalocalcaneal fusion with a unique combination of hindfoot nail and trabecular metal cone construct in a single procedure is a successful option for the treatment of large, recurrent GCT lesions in the distal tibia.

## Introduction

Giant cell tumors (GCTs) of bone are benign, locally aggressive neoplasms most commonly involving the epiphyses of the distal femur, proximal tibia, and distal radius [1]. Reconstruction options for symptomatic lesions are challenging due to severe bone loss, cortical destruction, and high recurrence rates. GCTs involving the foot and ankle are rare, comprising less than 4% of all GCTs of bone [2,3]. Few prior case reports have described success with excision, curettage, and cementation [4-7]. Here we discuss the case of a unique, large, recurrent GCT in the distal tibia metadiaphysis with severe adjacent tibiotalar joint degeneration, originally thought to be an aneurysmal bone cyst (ABC), that was refractory to two prior excisions and curettages performed at an outside hospital. The patient was successfully treated with extended curettage, argon beam coagulation, peroxide lavage, and tibiotalocalcaneal (TTC) arthrodesis using an intramedullary hindfoot fusion nail in conjunction with trabecular metal implant reconstruction and surrounding autograft, allograft, and cementation. 

## Case presentation

A 36-year-old female status post two prior curettage and grafting procedures five and seven years prior presented with acutely increased pain and swelling of the left ankle over the previous five months. She was otherwise healthy and eight months pregnant at the initial presentation. Pain was worsened with activity and refractory to nonoperative treatment including physical therapy, bracing, activity modification, and over-the-counter pain medications. Pathology reports from her prior surgeries were consistent with an ABC.

On physical examination, she had a prominent, firm mass over the left distal leg and ankle that was tender to palpation. She had a healed anterolateral incision with peri-incisional numbness. Her ankle range of motion was extremely limited, with pain throughout any attempted motion. Motor strength and sensation were intact throughout the foot and ankle. She had no other systemic symptoms.

**Figure 1:**
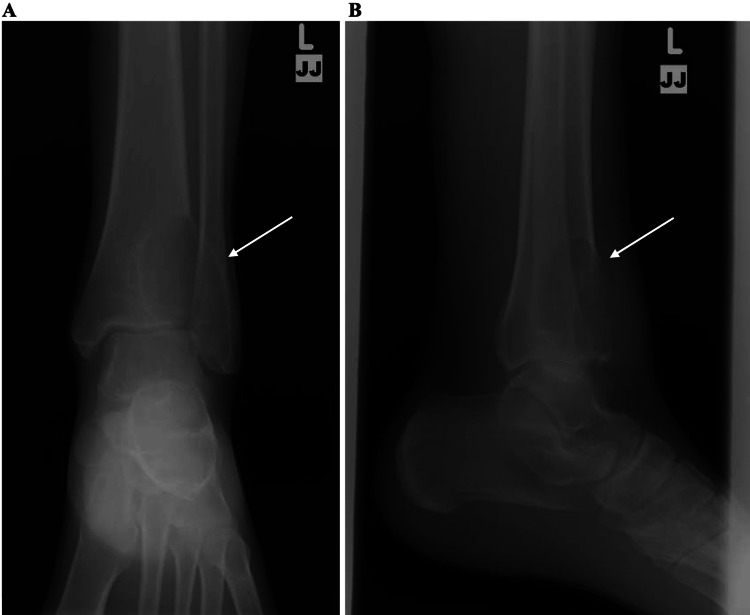
Initial x-rays AP (A) and lateral (B) x-ray views of the left ankle of the patient when she initially presented to the clinic. The lesion is marked with arrows.

**Figure 2:**
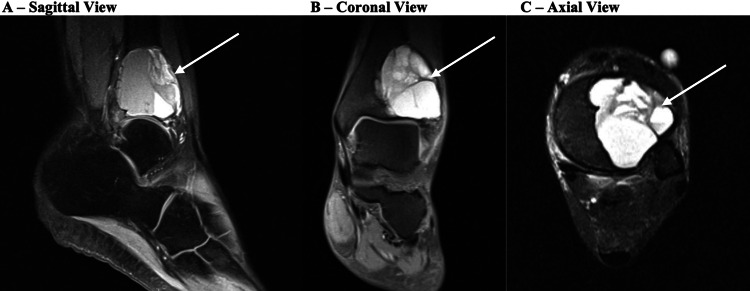
Prior MRI Sagittal (A), coronal (B), and axial (C) MRI views of the left ankle of the patient prior to her previous surgeries. The lesion is marked with arrows.

Plain radiographs at her initial presentation showed a large expansile lytic lesion encompassing nearly the entire distal tibia with cortical expansion and extending laterally into the fibula (Figures 1A, 1B). MRI images obtained before her previous surgeries showed a well-defined cystic lesion in the distal tibia with fluid levels present and lateral cortex expansion, measuring approximately 3.8 cm long in coronal plane x 3.3 cm wide in sagittal plane x 3.6 cm deep in axial plane from seven years prior, and approximately 6 cm x 3.8 cm x 4.4 cm on recurrence five years prior, with no significant periosteal reaction or sclerosis (Figures 2A-2C). The plan was for the patient to undergo a repeat MRI with contrast postpartum. 

**Figure 3:**
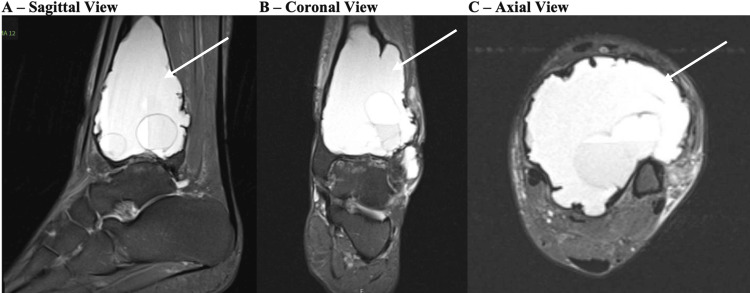
Repeat MRI Sagittal (A), coronal (B), and axial (C) MRI views of the left ankle of the patient when she returned to the clinic several months later. The lesion is marked with arrows.

The patient re-presented seven months postpartum at which point her pain had continued to worsen. Her physical examination and repeat plain radiographs were unchanged. MRI showed a lytic and cystic expansion of the distal tibia without aggressive periosteal reaction, measuring approximately 8.3 x 5.6 x 5.9 cm with fluid-fluid levels and internal septation (Figures 3A-3C), and severe erosive changes at both the tibial plafond and talar dome with multiple areas of full-thickness articular cartilage loss and subchondral cystic changes. A repeat biopsy was performed by Interventional Radiology, with results consistent with ABC showing histiocytes, rare giant cells, few inflammatory cells, and numerous red blood cells. 

Surgical options were discussed with the patient. The first option was an isolated repeat lesion curettage and reconstruction with cement grafting. The second option was a single surgery that would include lesion curettage, reconstruction, and concurrent ankle and subtalar joint arthrodesis with an intramedullary nail to bypass the lesion and provide a load-sharing construct, given its size and location. This would concurrently address her symptomatic end-stage tibiotalar arthritis. The patient desired to minimize future surgeries and thus elected to proceed with the latter option.

**Figure 4:**
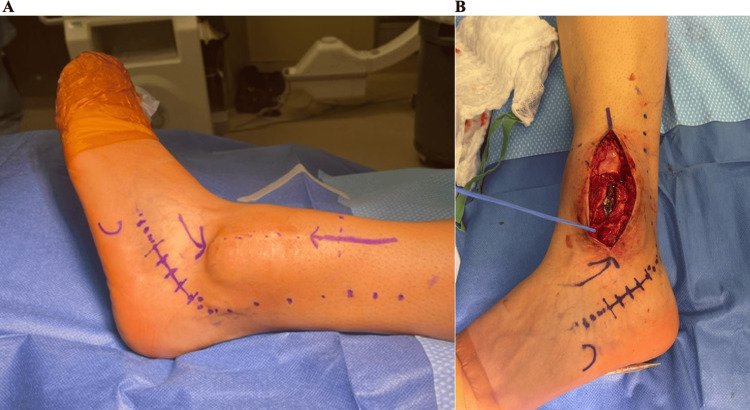
Intraoperative photos – initial approach and dissection (A) A marking pen was used to draw out the intended incision that would be used to isolate the lesion (marked with the two purple arrows). The lower dotted line was used for the ankle fusion portion of the procedure. (B) The initial dissection down towards the lesion. The blue vessel loop was used to isolate and protect the superficial peroneal nerve.

**Figure 5:**
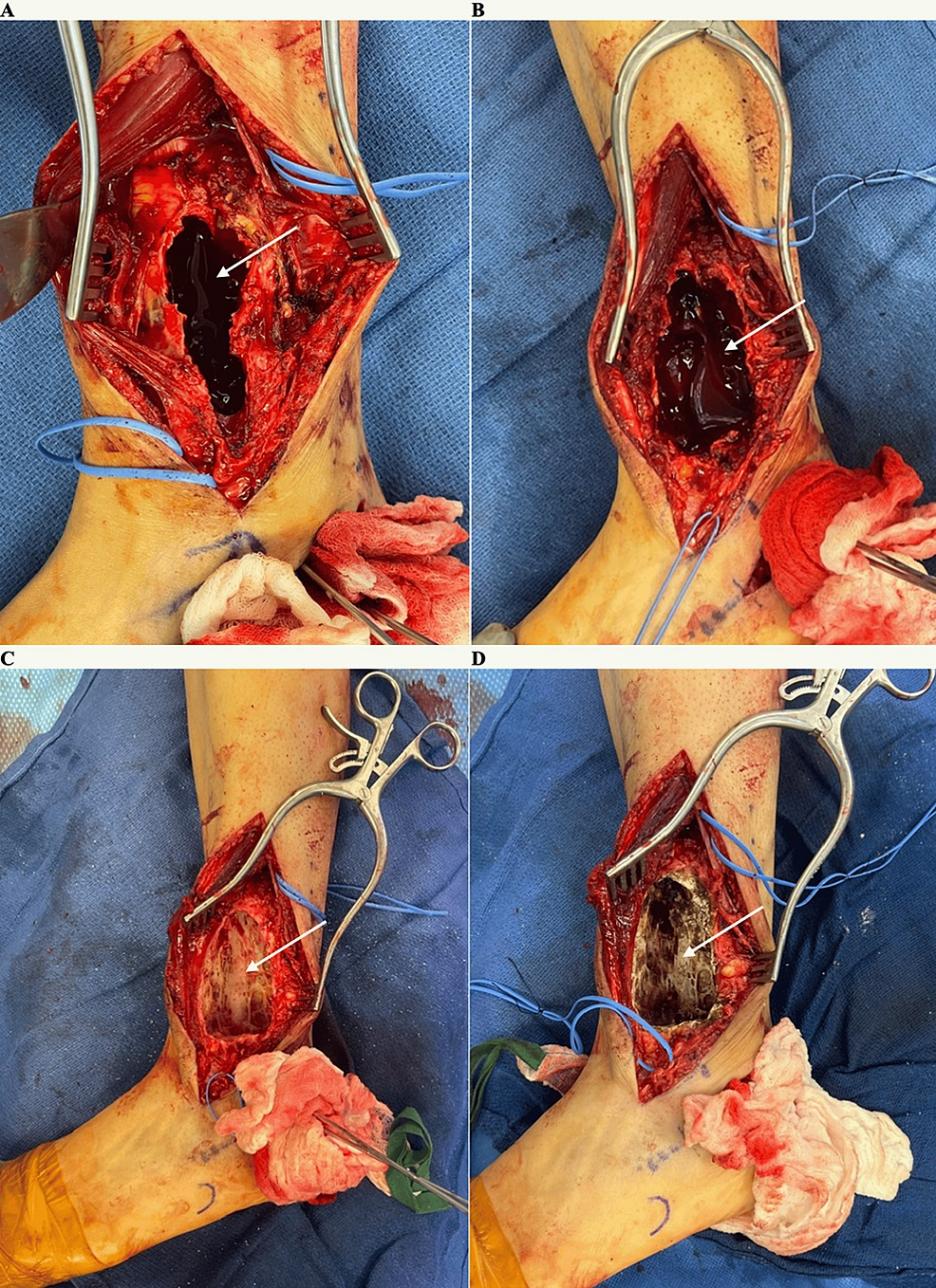
Intraoperative photos – evacuation and treatment of the lesion (A) The lesion was isolated and opened. An arrow indicates the inside of the lesion. (B) Samples of the serosanguinous core of the lesion (indicated with an arrow) were sent for intraoperative frozen pathology. (C) The lesion after being burned out and irrigated with peroxide. An arrow indicates the inside of the lesion. (D) The lesion after being treated with argon. An arrow indicates the inner surface of the lesion.

During surgery, the patient was positioned supine without a tourniquet. Her previous anterolateral incision was utilized with extension proximally and distally with dissection down to the lesion (Figures 4A, 4B). The superficial peroneal nerve was identified and protected throughout the operation. The bone was extremely soft and thin, with minimal anterior and lateral tibial cortex remaining. A rongeur was used to open the bone, and samples of the mostly serosanginous fluid and solid tissue from the edge of the lesion were sent for intraoperative frozen pathology. Intraoperative pathology analysis was again consistent with the previous diagnosis of ABC with no malignant cells identified. The biopsy was followed by extended curettage of the lesion confirmed radiographically with C-arm, burring of the cyst, irrigation with peroxide, and argon treatment as local adjuvant to decrease recurrence risk (Figures 5A-5D). 

Zimmer trabecular metal implants of appropriate size were trialed for the reconstruction of the void. Through the same approach, the distal tibial metaphysis was visualized, with essentially no tibial plafond articular surface remaining after lesion removal, consistent with preoperative imaging. The little remaining articular cartilage was removed, and the tibiotalar joint was prepared for arthrodesis in standard fashion using a combination of curettes, curved small osteotomes, and perforating drills.

**Figure 6:**
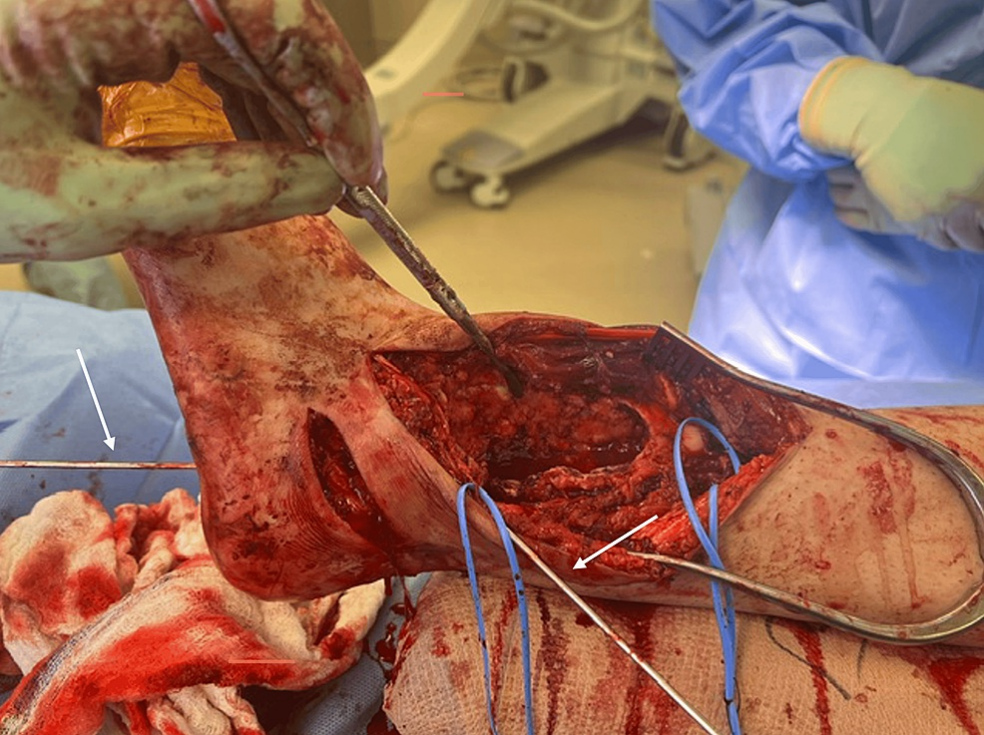
Intraoperative photos – K-wire fixation K-wires (indicated with arrows) were used to hold provisional fixation of the ankle.

**Figure 7:**
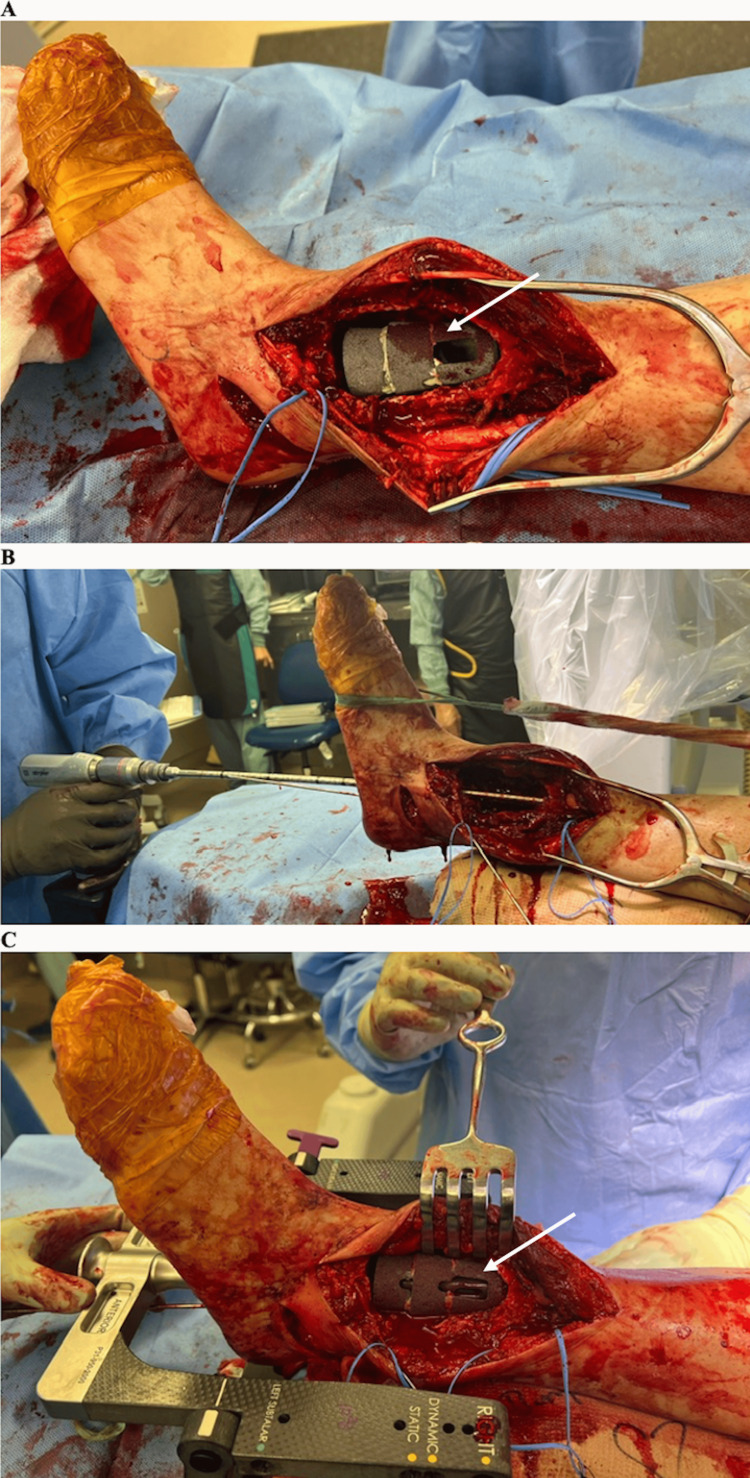
Intraoperative photos – metal cone implants and passage of nail (A) An arrow indicates the trabecular metal cone placed inside the hollowed-out lesion in the tibia. (B) A reamer was used to ream out the path of the eventual tibiotalocalcaneal nail. (C) The 11.5x250mm straight hindfoot fusion nail was passed through the calcaneus and talus and into the tibia, through the trabecular metal cone. The arrow indicates where the nail is passing through the cone.

**Figure 8:**
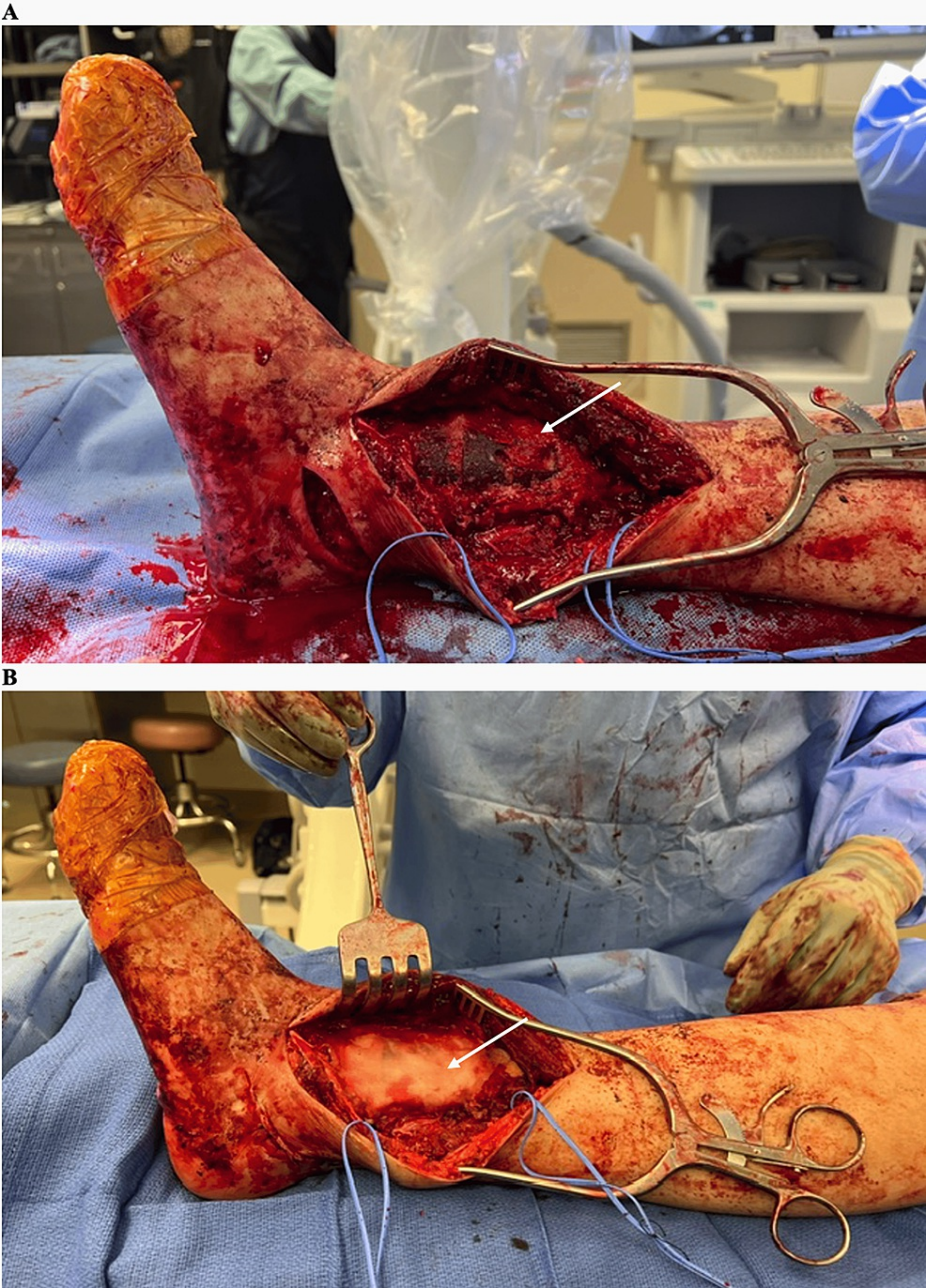
Intraoperative photos – bone graft and bone cement coverage and closure (A) Autograft, allograft, and demineralized bone matrix (marked with an arrow) were used to fill in the residual defect surrounding the trabecular metal cone and nail. (B) Bone cement with tobramycin (marked with an arrow) was used to recreate the cortices of the tibia prior to closure.

A separate 4cm standard sinus tarsi incision was made, and subtalar arthrodesis was performed via standard technique. Allograft was prepared according to manufacturer instructions and distributed between the ankle and subtalar joints to augment arthrodesis. The Achilles tendon was noted to be tight, and therefore a triple hemisection Achilles lengthening procedure was performed using three percutaneous incisions. Next, the fibula was exposed through the same incision. An osteotomy was performed to remove approximately 11 cm of distal fibula for autograft within the fusions and to fill the remaining void around the implants at the lesion site. The talus was then reduced on top of the calcaneus, followed by ankle joint reduction confirmed clinically and under multiplanar fluoroscopy. Large K-wires were used for provisional fixation (Figure 6). The calcaneus, talus, and tibia were sequentially reamed from an 8mm reamer to a final size of 12.5mm when appropriate bony chatter was obtained. An 11.5 x 250 mm straight hindfoot fusion nail was placed through the 3cm plantar incision. Before passage of the nail up the tibia, metal trabecular cones of appropriate size were placed into the void that had been filled by the lesion, stacked, and cemented together. The fusion nail was then advanced through the stabilized trabecular metal construct up the tibia (Figures 7A-7C). The nail was then secured using distal and proximal interlock screws per standard technique. Fibular autograft, cancellous allograft, and demineralized bone matrix were used to fill residual defects surrounding the nail and trabecular cone construct. Bone cement with tobramycin was then used to recreate the lateral and anterior distal tibial cortices (Figures 8A, 8B). The wounds were then closed in a layered fashion and dressed in a sterile manner, and a posterior mold sugar tong splint was applied. Postoperatively she was maintained non-weight bearing for 12 weeks and placed on aspirin 81mg twice daily for four weeks for DVT prophylaxis. CT scan of the ankle performed at 12 weeks postoperatively demonstrated appropriate consolidation of the ankle and subtalar fusion sites, progressive weight bearing, and physical therapy were initiated at this point.

Despite the previous biopsy results being consistent with ABC, the final intra-operative pathology resulted in a diagnosis of a GCT. The plan then became to proceed with postoperative monitoring protocol for GCTs of bone, including advanced imaging every three months and yearly chest imaging to rule out pulmonary metastases. CT chest showed no evidence of metastasis.

**Figure 9:**
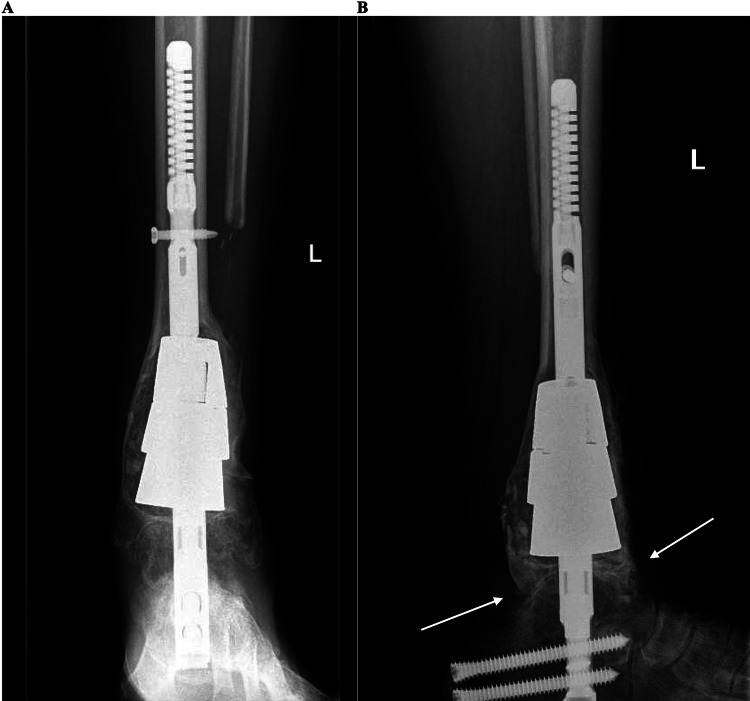
Postoperative x-rays AP (A) and lateral (B) x-ray views of the left ankle with trabecular metal cones and tibiotalocalcaneal nail in place six months postoperatively. The tibiotalar fusion site is marked with arrows in (B).

By 6 months postoperatively, she could ambulate using the assistance of a front wheel walker with minimal pain and swelling. She experienced minimal pain with palpation of the ankle. She had no movement at the ankle and subtalar joint, consistent with fusion. X-rays showed consolidated subtalar and ankle joint fusions without evidence of lesion recurrence (Figures 9A, 9B). 

## Discussion

This was a case of a recurrent, large GCT in the distal tibia, originally thought to be an ABC, successfully treated with extended curettage and reconstruction with TTC nail and trabecular metal implants by a fellowship-trained foot and ankle surgeon and an orthopedic oncologist. The unique aspects of this case include the location and size of the lesion, multiple recurrences despite prior resection and curettage, and the method and combination of surgical procedures during our reconstruction.

GCTs are approximately 5% of all primary bone tumors and 20% of benign tumors analyzed with biopsy [8]. Most lesions present in the third and fourth decades of life [8]. They are most commonly found in meta-epiphyseal lesions of long bones [8]. 50% of GCTs occur around the knee followed by the distal radius and the sacrum [8]. Less than 2% of GCTs occur in the foot and ankle [9-11]. The large size, proximity of the lesion to the ankle joint, and severe diffuse degenerative joint changes created the need for a well-planned multidisciplinary approach to surgical management. Additionally, the multiple recurrences following previous surgeries illustrated the need for more aggressive treatment. 

Typically, these lesions are treated with intralesional curettage to preserve anatomy as most lesions are near a joint and benign [12,13]. After curettage, these areas can be augmented with bone grafting, with or without adjuvant treatments such as argon beam coagulation, phenol, cauterization, cryotherapy with liquid nitrogen, or hydrogen peroxide [14-17]. Wide resections have been shown to have decreased local recurrence rate when compared to intralesional curettage from around 73 to near 100%, however, it is more invasive [18]. 

Surgical options for this patient included repeat curettage, grafting, and cement augmentation with isolated ankle fusion or concurrent ankle and subtalar fusion. Given the size of this lesion, it was preferred to utilize a load-sharing implant concurrently with trabecular metal cones to fill the residual large void and sufficiently bypass the area both proximally and distally. 

One concern was whether successful fusion would be possible given the extent of bony resection necessary. Prior studies have shown a minimum of 25%-49% of osseous bridging produces improved clinical outcomes [19]. Despite the size of the lesion and extent of grafting required, this patient still had a successful bony fusion with sufficient osseous bridging with clinical functional improvement. 

An alternative construct to consider would have been a custom 3D-printed implant. However, this poses its own challenges, as the final dimensions post-lesion resection cannot be perfectly predicted preoperatively. Additionally, this requires significant planning for implant turnaround time, as well as incurs higher costs [20]. However, a custom implant may have allowed for less grafting and cementation. 

## Conclusions

While rare, GCTs can be very painful and can significantly affect the quality of life of patients. Current treatment options involve resection and cementation of lesions. However, these procedures are limited and difficult, especially in load-bearing parts of the skeleton such as the ankle, as resection can significantly affect the structural integrity of the bone and lesions often recur despite surgical treatment. Although more involved than simple curettage and cementation, combined extended curettage, reconstruction, and TTC fusion with a unique combination of hindfoot nail and trabecular metal cone construct in a single procedure is a successful option for the treatment of large recurrent GCT lesions in the distal tibia. It allows for a more complete resection of the lesion, a lower risk of recurrence, and a more stable construct that allows for better function postoperatively.
